# Supplementary material for: Association of Dietary Intakes and Genetically Determined Serum Concentrations of Mono and Poly Unsaturated Fatty Acids on Chronic Kidney Disease: Insights from Dietary Analysis and Mendelian Randomization
Source: Nutrients. 2022 Mar 15;14(6):1231. doi: 10.3390/nu14061231 (PMC8954914; doi:10.3390/nu14061231)
Supplement: Supplementary file 1 [file nutrients-14-01231-s001.zip › nutrients-1596452-supplementary.pdf]

Supplementary Materials:

Table S1. Summary results of the genetic loci of monounsaturated fatty acids (MUFAs) and polyunsaturated fatty acids (PUFAs)

Traits	SNP	GX	GX SE	EA	OA	EAF
Heptadecenoate	rs220992	0.0155	0.0033	A	G	0.4501
	rs1822906	0.0188	0.0041	A	G	0.205
	rs4288115	-0.0172	0.0038	A	T	0.3119
	rs10098688	0.0242	0.0054	A	C	0.3373
	rs10258925	0.0153	0.0034	A	G	0.2709
	rs11118120	-0.0209	0.0047	A	C	0.8498
Myristoleate	rs677603	0.0224	0.004	T	C	0.2536
	rs11728793	-0.021	0.0042	T	C	0.7845
	rs9888349	-0.0192	0.004	T	C	0.7742
Oleate	rs11719883	-0.0191	0.0034	C	G	0.8444
	rs6453591	0.0133	0.0025	A	T	0.771
	rs11133892	0.012	0.0023	A	G	0.4423
	rs13019537	0.0157	0.0032	C	G	0.841
Palmitoleate	rs677603	0.022	0.0042	T	C	0.2538
	rs11728793	-0.0215	0.0044	T	C	0.7844
	rs13019537	0.0228	0.005	C	G	0.8416
Alpha-Linolenic acid	rs509360	-0.011	0.0011	A	G	0.3293
	rs174468	-0.0076	0.0011	A	G	0.4291
	rs2453710	0.005	9.00E-04	A	G	0.5445
Eicosapentaenoic acid	rs1145652	0.0356	0.0066	A	G	0.8653
	rs2269928	0.0639	0.0082	T	G	0.7872
	rs2727270	-0.0762	0.007	T	C	0.1081
	rs2521572	-0.0614	0.0108	T	G	0.0531

EA: effect allele; OA: other allele, EAF: effect allele frequency; GX: the per-allele effect on standard deviation units of the adrenic acid; GX SE: standard error of GX.

<table>
<tr><th colspan="11">Table S2: Results of the Mendelian Randomization (MR) analysis for 10-heptadecenoate (17:1) and kidney function</th></tr>
<tr><th colspan="2" rowspan="2">Exposures</th><th colspan="4">MR</th><th colspan="3">Heterogeneity</th><th colspan="3">Pleiotropy</th></tr>
<tr><th>Method</th><th>beta</th><th>SE</th><th>p</th><th>Method</th><th>Q</th><th>P-value</th><th>Intercept</th><th>SE</th><th>p</th></tr>
<tr><td colspan="2" rowspan="4">CKD</td><td>MR Egger</td><td>-1.452</td><td>2.159</td><td>0.5381</td><td rowspan="2">MR-Egger</td><td rowspan="2">4.018</td><td rowspan="2">0.403</td><td rowspan="4">0.032</td><td rowspan="4">0.041</td><td rowspan="4">0.479</td></tr>
<tr><td>WM</td><td>0.1862</td><td>0.4959</td><td>0.7073</td></tr>
<tr><td>IVW</td><td>0.2052</td><td>0.3796</td><td>0.5888</td><td rowspan="2">IVW</td><td rowspan="2">4.635</td><td rowspan="2">0.469</td></tr>
<tr><td>RAPS</td><td>0.2507</td><td>0.4036</td><td>0.5345</td></tr>
<tr><td rowspan="12">eGFR</td><td rowspan="4">Total</td><td>MR Egger</td><td>-0.1917</td><td>0.1246</td><td>0.1988</td><td rowspan="2">MR-Egger</td><td rowspan="2">2.49</td><td rowspan="2">0.635</td><td rowspan="4">0.0035</td><td rowspan="4">0.0023</td><td rowspan="4">0.212</td></tr>
<tr><td>WM</td><td>-0.01484</td><td>0.02864</td><td>0.6044</td></tr>
<tr><td>IVW</td><td>-0.0098</td><td>0.02205</td><td>0.6569</td><td rowspan="2">IVW</td><td rowspan="2">4.69</td><td rowspan="2">0.452</td></tr>
<tr><td>RAPS</td><td>-0.01245</td><td>0.02379</td><td>0.6007</td></tr>
<tr><td rowspan="4">Non-DM</td><td>MR Egger</td><td>-0.1643</td><td>0.1246</td><td>0.2577</td><td rowspan="2">MR-Egger</td><td rowspan="2">1.895</td><td rowspan="2">0.742</td><td rowspan="4">0.0031</td><td rowspan="4">0.0023</td><td rowspan="4">0.256</td></tr>
<tr><td>WM</td><td>0.003538</td><td>0.02883</td><td>0.9023</td></tr>
<tr><td>IVW</td><td>-0.00174</td><td>0.02205</td><td>0.937</td><td rowspan="2">IVW</td><td rowspan="2">3.623</td><td rowspan="2">0.631</td></tr>
<tr><td>RAPS</td><td>-0.0018</td><td>0.0236</td><td>0.9391</td></tr>
<tr><td rowspan="4">DM</td><td>MR Egger</td><td>-0.3324</td><td>0.5044</td><td>0.546</td><td rowspan="2">MR-Egger</td><td rowspan="2">3.041</td><td rowspan="2">0.551</td><td rowspan="4">0.0063</td><td rowspan="4">0.0095</td><td rowspan="4">0.546</td></tr>
<tr><td>WM</td><td>-0.03689</td><td>0.1174</td><td>0.7533</td></tr>
<tr><td>IVW</td><td>-0.00512</td><td>0.08919</td><td>0.9542</td><td rowspan="2">IVW</td><td rowspan="2">3.476</td><td rowspan="2">0.632</td></tr>
<tr><td>RAPS</td><td>-0.00529</td><td>0.09403</td><td>0.9552</td></tr>
<tr><td colspan="11">WM: Weighted median, IVW: Inverse variance weighted, SE: standard error, beta: beta-coefficients, MR: Mendelian randomization, CKD: chronic kidney disease, eGFR: estimated glomerular filtration rate, DM: type 2 diabetes mellitus.</td></tr>
</table>

Table S3: Results of the Mendelian Randomization (MR) analysis for Myristoleic acid (14:1) and kidney function

Exposures		MR				Heterogeneity			Pleiotropy		
		Method	beta	SE	p	Method	Q	P-value	Intercept	SE	p
CKD		MR Egger	1.516	7.934	0.8798	MR-Egger	0.006	0.963	-0.019	0.17	0.929
		WM	0.5822	0.5844	0.3191						
		IVW	0.6265	0.4971	0.2075	IVW	0.019	0.932			
		RAPS	0.6267	0.5152	0.2238						
eGFR	Total	MR Egger	1.051	0.4409	0.2529	MR-Egger	0.158	0.639	-0.022	0.0092	0.252
		WM	0.000167	0.03946	0.9966						
		IVW	0.000189	0.04869	0.9969	IVW	5.863	0.069			
		RAPS	-0.00029	0.04657	0.995						
	Non-DM	MR Egger	1.065	0.4611	0.26	MR-Egger	0.256	0.615	-0.023	0.0096	0.252
		WM	-0.03421	0.0419	0.4143						
		IVW	-0.03424	0.05096	0.5016	IVW	5.932	0.063			
		RAPS	-0.03217	0.04877	0.5094						
	DM	MR Egger	**1.202**	**1.852**	**0.6334**	MR-Egger	0.136	0.712	-0.023	0.039	0.663
		WM	**0.1497**	**0.1462**	**0.3058**						
		IVW	**0.1106**	**0.1194**	**0.3542**	IVW	0.482	0.782			
		RAPS	**0.1113**	**0.124**	**0.3695**						

WM: Weighted median, IVW: Inverse variance weighted, SE: standard error, beta: beta-coefficients, MR: Mendelian randomization, CKD: chronic kidney disease, eGFR: estimated glomerular filtration rate, DM: type 2 diabetes mellitus.

Table S4: Results of the Mendelian Randomization (MR) analysis for Oleic acid (18:1) and kidney function

Exposures		MR				Heterogeneity			Pleiotropy		
		**Method**	**beta**	**SE**	**p**	**Method**	**Q**	**P-value**	**Intercept**	**SE**	**p**
**CKD**		**MR Egger**	2.875	3.562	0.5043	**MR-Egger**	2.14	0.346	**-0.04**	**0.052**	**0.518**
		**WM**	0.4119	0.7962	0.6049						
		**IVW**	0.1513	0.6296	0.8101	**IVW**	2.72	0.432			
		**RAPS**	0.169	0.6848	0.8051						
**eGFR**	**Total**	**MR Egger**	-0.1092	0.202	0.6429	**MR-Egger**	1.236	0.536	**0.0022**	**0.003**	**0.532**
		**WM**	0.02474	0.04365	0.5709						
		**IVW**	0.0397	0.036	0.2702	**IVW**	1.813	0.612			
		**RAPS**	0.0404	0.03787	0.2862						
	**Non-DM**	**MR Egger**	-0.04269	0.2139	0.8602	**MR-Egger**	0.382	0.823	**0.0015**	**0.0031**	0.678
		**WM**	0.05319	0.04456	0.2326						
		**IVW**	0.05862	0.0384	0.1269	**IVW**	0.612	0.892			
		**RAPS**	0.05896	0.04008	0.1413						
	**DM**	**MR Egger**	0.08902	1.56	0.9597	**MR-Egger**	6.923	0.063	**-0.0034**	**0.023**	0.869
		**WM**	-0.00945	0.2006	0.9624						
		**IVW**	-0.1402	0.229	0.5404	**IVW**	7.325	0.075			
		**RAPS**	-0.1282	0.2189	0.5581						

WM: Weighted median, IVW: Inverse variance weighted, SE: standard error, beta: beta-coefficients, MR: Mendelian randomization, CKD: chronic kidney disease, eGFR: estimated glomerular filtration rate, DM: type 2 diabetes mellitus.

Table S5: Results of the Mendelian Randomization (MR) analysis for Palmitoleic acid (16:1) and kidney function											
Exposures		MR				Heterogeneity			Pleiotropy		
		Method	beta	SE	p	Method	Q	P-value	Intercept	SE	p
**CKD**		**MR Egger**	-5.283	21.86	0.849	**MR-Egger**	0.08346	0.772	**0.13**	**0.48**	**0.834**
		**WM**	0.5807	0.5993	0.3325						
		**IVW**	0.5338	0.5003	0.286	**IVW**	0.1543	0.925			
		**RAPS**	0.5348	0.5187	0.3026						
**eGFR**	**Total**	**MR Egger**	1.27	1.261	0.4979	**MR-Egger**	1.02	0.311	**-0.027**	**0.028**	**0.509**
		**WM**	0.06664	0.03731	0.0741						
		**IVW**	0.04549	0.02855	0.111	**IVW**	1.98	0.371			
		**RAPS**	0.04663	0.03082	0.1303						
	**Non-DM**	**MR Egger**	1.798	1.309	0.4006	**MR-Egger**	0.85	0.356	**-0.039**	**0.029**	**0.404**
		**WM**	0.04409	0.03972	0.267						
		**IVW**	0.02105	0.03516	0.5493	**IVW**	2.69	0.265			
		**RAPS**	0.02222	0.03453	0.5199						
	**DM**	**MR Egger**	-5.085	5.266	0.5111	**MR-Egger**	0.14	0.701	**0.11**	**0.12**	**0.506**
		**WM**	0.1552	0.1495	0.2991						
		**IVW**	0.08405	0.1202	0.4842	**IVW**	1.08	0.574			
		**RAPS**	0.08535	0.126	0.4982						

WM: Weighted median, IVW: Inverse variance weighted, SE: standard error, beta: beta-coefficients, MR: Mendelian randomization, CKD: chronic kidney disease, eGFR: estimated glomerular filtration rate, DM: type 2 diabetes mellitus.

Table S6: Results of the Mendelian Randomization (MR) analysis for alpha-linolenic acid (18:3) and kidney function

Exposures		MR				Heterogeneity			Pleiotropy		
		Method	beta	SE	p	Method	Q	P-value	Intercept	SE	p
CKD		MR Egger	-5.851	3.779	0.365	MR-Egger	0.869	0.356	0.052	0.03	0.333
		WM	0.5576	1.325	0.674						
		IVW	0.3791	1.598	0.8125	IVW	3.265	0.142			
		RAPS	0.3165	1.484	0.8311						
eGFR	Total	MR Egger	0.04804	0.2264	0.8669	MR-Egger	0.093	0.752	-0.00082	0.0018	0.732
		WM	-0.03475	0.0784	0.6576						
		IVW	-0.04827	0.06929	0.486	IVW	0.236	0.869			
		RAPS	-0.04835	0.07129	0.4977						
	Non-DM	MR Egger	0.1447	0.2264	0.638	MR-Egger	0.695	0.402	-0.0015	0.0018	0.565
		WM	-0.00868	0.07865	0.9121						
		IVW	-0.03081	0.06929	0.6565	IVW	1.365	0.503			
		RAPS	-0.03102	0.07169	0.6652						
	DM	MR Egger	-0.4703	0.9203	0.6993	MR-Egger	0.050	0.823	0.00061	0.0074	0.948
		WM	-0.4305	0.2997	0.1509						
		IVW	-0.3987	0.2833	0.1593	IVW	0.057	0.974			
		RAPS	-0.3988	0.2924	0.1725						

WM: Weighted median, IVW: Inverse variance weighted, SE: standard error, beta: beta-coefficients, MR: Mendelian randomization, CKD: chronic kidney disease, eGFR: estimated glomerular filtration rate, DM: type 2 diabetes mellitus.

**Table S7: Results of the Mendelian Randomization (MR) analysis for Eicosapentaenoic acid (20:5) and kidney function**												
**Exposures**		**MR**				**Heterogeneity**			**Pleiotropy**			
		**Method**	**beta**	**SE**	**p**	**Method**	**Q**	**P-value**	**Intercept**	**SE**	**p**
**CKD**		**MR Egger**	0.248	0.79	0.7833	**MR-Egger**	0.81	0.666	-0.015	**0.048**	**0.78**
		**WM**	0.06092	0.2358	0.7961						
		**IVW**	0.004894	0.2042	0.9809	**IVW**	0.91	0.832			
		**RAPS**	0.004911	0.2104	0.9814						
**eGFR**	**Total**	**MR Egger**	0.006322	0.06748	0.9339	**MR-Egger**	4.695	0.098	0.00041	**0.0041**	**0.93**
		**WM**	-0.00053	0.01638	0.9744						
		**IVW**	0.01276	0.01443	0.3762	**IVW**	4.723	0.625			
		**RAPS**	0.01192	0.01471	0.4176						
	**Non-DM**	**MR Egger**	-0.01615	0.07769	0.8545	**MR-Egger**	5.368	0.075	0.0015	0.0048	0.789
		**WM**	0.001395	0.01758	0.9367						
		**IVW**	0.006813	0.01652	0.68	**IVW**	5.692	0.653			
		**RAPS**	0.005785	0.01705	0.7343						
	**DM**	**MR Egger**	0.2331	0.1875	0.3398	**MR-Egger**	0.389	0.823	**-0.013**	**0.011**	**0.364**
		**WM**	0.04069	0.05736	0.4781						
		**IVW**	0.02242	0.04945	0.6502	**IVW**	1.785	0.623			
		**RAPS**	0.02257	0.0512	0.6593						

WM: Weighted median, IVW: Inverse variance weighted, SE: standard error, beta: beta-coefficients, MR: Mendelian randomization, CKD: chronic kidney disease, eGFR: estimated glomerular filtration rate, DM: type 2 diabetes mellitus.
